# Applications of Microbial Organophosphate-Degrading Enzymes to Detoxification of Organophosphorous Compounds for Medical Countermeasures against Poisoning and Environmental Remediation

**DOI:** 10.3390/ijms25147822

**Published:** 2024-07-17

**Authors:** Tatiana Pashirova, Rym Salah-Tazdaït, Djaber Tazdaït, Patrick Masson

**Affiliations:** 1 Institute of Fundamental Medicine and Biology, Kazan Federal University, 18 Kremlyovskaya St., 420008 Kazan, Russia; pashirova@iopc.ru; 2 Arbuzov Institute of Organic and Physical Chemistry, FRC Kazan Scientific Center of RAS, Arbuzov Str. 8, 420088 Kazan, Russia; 3 Bioengineering and Process Engineering Laboratory (BIOGEP), National Polytechnic School, 10 Rue des Frères Oudek, El Harrach, Algiers 16200, Algeria; rym.salah@g.enp.edu.dz (R.S.-T.); d.tazdait@univ-alger.dz (D.T.); 4 Department of Nature and Life Sciences, University of Algiers, Benyoucef Benkhedda, 2 Rue Didouche Mourad, Algiers 16000, Algeria

**Keywords:** bioscavenger, bioremediation, cholinesterase, enzymotherapy, micro-organisms, organophosphorous, phosphotriesterase

## Abstract

Mining of organophosphorous (OPs)-degrading bacterial enzymes in collections of known bacterial strains and in natural biotopes are important research fields that lead to the isolation of novel OP-degrading enzymes. Then, implementation of strategies and methods of protein engineering and nanobiotechnology allow large-scale production of enzymes, displaying improved catalytic properties for medical uses and protection of the environment. For medical applications, the enzyme formulations must be stable in the bloodstream and upon storage and not susceptible to induce iatrogenic effects. This, in particular, includes the nanoencapsulation of bioscavengers of bacterial origin. In the application field of bioremediation, these enzymes play a crucial role in environmental cleanup by initiating the degradation of OPs, such as pesticides, in contaminated environments. In microbial cell configuration, these enzymes can break down chemical bonds of OPs and usually convert them into less toxic metabolites through a biotransformation process or contribute to their complete mineralization. In their purified state, they exhibit higher pollutant degradation efficiencies and the ability to operate under different environmental conditions. Thus, this review provides a clear overview of the current knowledge about applications of OP-reacting enzymes. It presents research works focusing on the use of these enzymes in various bioremediation strategies to mitigate environmental pollution and in medicine as alternative therapeutic means against OP poisoning.

## 1. Introduction

Organophosphorous compounds (OPs) are thio/oxo phosphoesters. They are highly toxic compounds widely used all over the word for multiple applications. They have been used for more than 70 years as pesticides [1], as drugs or pro-drugs in human and veterinary medicine [2], and as antiwear agents and flame retardants in industrial oils such as tricresyl phosphate [3]. In particular, this later compound involved in aerotoxic syndrome may also cause accidental or criminal poisoning, e.g., in the USA during the Prohibition due its presence in adulterated alcohols and in Morocco in oil of canned fish. However, the most toxic OPs are banned chemical warfare agents (CWA) [1] like G agents (tabun, sarin, cylclohexyl-sarin and soman), V agents and A agents, which are the so-called novichoks (“newcomers”). The latter compounds, about 10 times more toxic than VX and 10^4^ times more toxic than OP pesticides [4], are highly stable in the environment and treatment of poisoning is very difficult [5].

The use of pesticides in agriculture worldwide has increased significantly during the past three decades, passing from about 1.8 million tons in 1990 to around 3.5 million tons in 2021, of which more than 7.5 × 10^5^ tons are insecticides, which include OPs [6]. Because of their toxicity, OPs, including insecticides and nerve agents, pose significant threats to human health and the environment. These compounds can persist in the environment for long periods (up to 360 days) and can contaminate food products, soil, and water sources [7]. One promising approach to mitigating the risks associated with OPs is the use of a bioremediation technique for their degradation. Bioremediation is a cost-effective and environmentally friendly technique that utilizes micro-organisms (such as bacteria, fungi, and algae) or the enzymes they produce to degrade, transform, or remove contaminants from soil, water, or air. This process relies on the natural metabolic capabilities of these organisms to break down complex pollutants into less harmful substances [8,9]. Many studies have reported the efficiency of using micro-organisms, such as algae, fungi, and bacteria, to degrade the complex chemical structures of OPs into simpler and less toxic molecules through different enzymatic processes [10,11,12,13,14]. However, microbial degradation may be challenging to apply effectively in real environmental conditions due to several limitations: (1) the degradation process can be slow, taking a considerable time (from several weeks to several months) for the micro-organisms to degrade the pesticides completely; (2) microbial populations can show genetic instability, leading to differences in their degradation abilities; (3) less effectiveness in degrading certain types of pesticides with complex chemical structures; and (4) microbial activity can be affected by several environmental factors such as pesticide bioavailability, humidity, pH, salinity, and temperature, whose fluctuations may reduce degradation effectiveness [15,16]. To overcome these constraints, the use of purified degrading enzymes offers a number of advantages over entire cell systems. These include the potential for specifically targeting organic pollutants with a higher speed of degradation and efficiency, the innocuity of the process, which does not produce any risky by-products, unlike microbial processes, and the ability to perform in a variety of environmental situations [17]. Furthermore, this approach can be used in large-scale bioremediation strategies, such as in situ and ex situ biodegradation of OPs, offering sustainable and cost-effective solutions to environmental pollution.

Owing to the high risk of accidental and self-poisoning due to the use of OPs in agriculture, the threat of implementation of OPs in terrorist acts and in asymmetric conflicts, and environmental consequences of the extensive use of OPs in the world, it was important to review new approaches for the decontamination, remediation, and therapeutic means against these compounds. Several reviews about the use of OP-degrading enzymes in the fields of medicine and bioremediation were published in the past few years [14,18,19,20,21]. These fields are now so important that while our manuscript was under review, an article covering the same topics was published [22]. Thus, this review updates our knowledge, exposes basic concepts and problems, and explores the effectiveness of using OP-degrading enzymes from micro-organisms in prophylaxis and treatment of OP poisoning and treating OP contamination for sustainable environmental management. Furthermore, the promising prospect of employing enzyme-containing nanoparticles for medical purposes and to mitigate OP contamination of actual and synthetic aqueous effluents are also explored and recent achievements are reported.

## 2. Toxicity of OPs

OPs are potent irreversible inhibitors of serine hydrolases [23], including esterases (carboxylesterases, lipases), acylamidases, and proteases. The most important enzymes targeted by OPs are cholinesterases (ChE).

Acetylcholinesterase (AChE; EC. 3.1.1.7) plays a major role in the cholinergic system in terminating the action of the neurotransmitter acetylcholine. The related enzyme butyrylcholinesterase (BChE; EC.3.1.1.8) has a minor role in cholinergic system and its physiological functions are not well known, but it is of importance in pharmacology and toxicology in the degradation of drugs and scavenging OP and carbamate toxicants [2].

**Figure 1:**
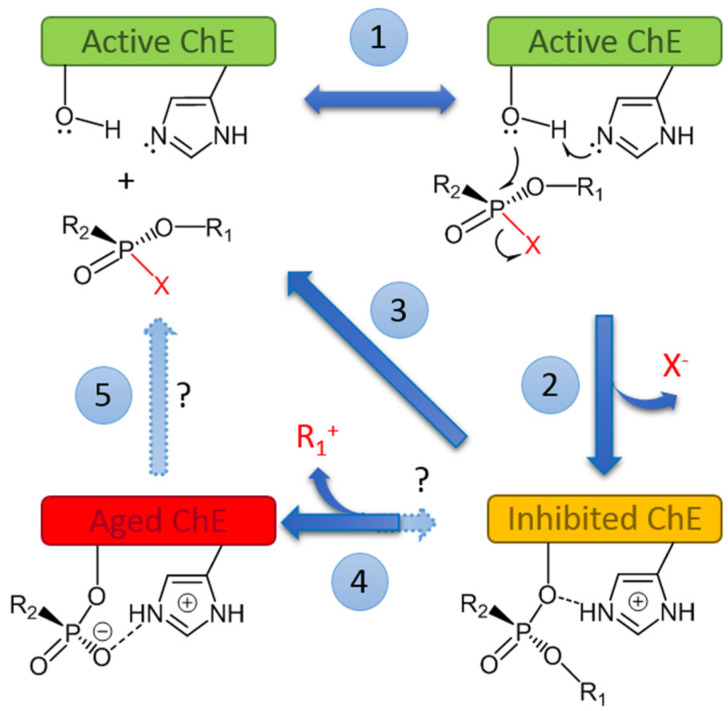
Mechanism of inhibition, aging, and reactivation of cholinesterases (ChEs) by OPs. Two key residues in active center of ChEs are depicted: the catalytic serine (-Ö-H) and the catalytic histidine.

Figure 1 describes the minimum reaction scheme of ChE inhibition by OPs, post-inhibition and reactivation. OP first binds reversibly to ChEs (step1). Then, after formation of this complex, the active site (serine, E-ÖH) is phosphylated. The phosphylation reaction is accompanied by release of the OP leaving group X- (step 2). X- can be a halide (F-) or an oxo/thio alkyl/aryl ion. It is important to note that unlike reactions of ChEs with ester substrates, water is too weak of a nucleophile for fast spontaneous reactivation of phosphylated ChEs. Thus, OPs can be regarded as pseudo-substrates of ChEs [2]. Therefore, phosphylated enzymes can only be reactivated in a short time by strong nucleophilic agents, like oximate ions used as antidotes in emergency treatment of acute OP poisoning [24,25] (step 3). Then, post-inhibitory reactions may complicate the scheme: the phosphyl-ChE conjugate may undergo a spontaneous dealkylation (step 4) through alkyl-oxygen bond scission (this reaction is called “aging”) [26]. Aging causes irreversible inactivation (non-reactivatability) of phosphylated enzyme. Aging can be very fast (t_1/2_ = 3 min at 37 °C for human AChE phosphonylated by soman, thus impairing practical reactivation in this case). The reactivation of aged ChEs has long been considered as a catch-22. However, drug-mediated reactivation of ChEs through the realkylation (+R_1_) of aged ChEs (reverse reaction, in step 4 and subsequent step 3, called “resurrection” or “resuscitation”) was recently demonstrated to be possible [27]. However, the direct displacement of the aged adduct (step 5) leading to spontaneous enzyme reactivation is still impossible, but research continues to solve this difficult issue (?).

Then, irreversible inhibition of ChEs by OPs leads to the accumulation of acetylcholine in synapses and a blockade of cholinergic transmissions. Actually, the inhibition of AChEs in peripheral (ganglia and neuromuscular junctions) and central nervous systems is the main cause of the acute toxicity of OPs [23]. This inhibition causes a major cholinergic syndrome. In addition, the irreversible inhibition of other hydrolases in the central nervous system and alkylation of different proteins plays a role in the sub-acute toxicity of OPs as well as in non-cholinergic toxicity, in particular long-term post-exposure effects [28]. Thus, phosphorylation of serine, tyrosine, lysine, and other residues in numerous proteins is also involved in the sub-lethal and chronic toxicity of OPs [29].

The medical counter measures of OP poisoning are imperfect. Prophylaxis means can partially mitigate the acute toxicity of OPs, oxime antidotal treatment of phosphylated ChEs does not work with certain OPs (either for steric reasons or due to fast aging reaction of phosphylated AChE), and symptomatic countermeasures are limited. Then, classical pharmacological approaches have reached their limits. Moreover, due to accumulation of OP molecules in depot sites (e.g., fat) and their subsequent slow release in the bloodstream, ChEs may remain inhibited for long periods of time. Therefore, the persistence of certain OPs in the body after exposure complicates treatments of acute poisoning. This has been known for a long time and is well documented for severe intoxications by parathion [30]. Yet, in the past 20 years, significant progress has been made in emergency pharmacological treatments of OP poisoning and medical management of chemical casualties [31,32,33,34,35,36].

The use of OP-reacting enzymes to trap, neutralize, and degrade OPs was initially proposed as an alternative to classical pharmacological means. It was based on the observation that several endogenous enzymes and OP-reacting proteins present in the skin, blood, and liver react with OPs and are involved in natural defenses against OP toxicity. Indeed, the presence of detoxifying or scavenging enzymes, such as BChE, in skin contributes to reduce the concentration of OP that penetrates into the body [37]. Liver enzymes play also an essential role in OP detoxification. In particular, glutathione S-transferases (GST, EC. 2.5.1.18) is involved in the degradation of alkyl/aryl chains of OPs [38], carboxylesterases (CaE; EC 3.1.1.1), other serine hydrolases, and irreversibly scavenge OPs after the phosphylation of their active site serine [39]. Lastly, blood bioscavengers significantly contribute to reduce the amount of OP molecules reaching neuro- and neuro-muscular targets. Plasma enzymes and OP-reacting proteins play a major role in this natural defense. In particular, paraoxonase-1 (PON-1, EC 3.1.8.1), an endogenous phosphotriesterase (PTE), may hydrolyze certain OPs at high rate. It is well known that animals in which the plasma concentration in PON-1 and/or in CaE is high, like rabbits, are relatively resistant to OPs [40]. Conversely, knockout mice for PON-1 are very sensitive to OPs [41]. However, unlike the plasma of most model animals, human plasma does not contain carboxylesterases [42,43]. However, human plasma contains BChE that effectively scavenges a fraction of OP molecules in the bloodstream. A special role is devoted to albumin. Albumin is the most abundant protein in plasma and lymph with a concentration close to 0.6 mM. It slowly reacts with esteryl-, carbamyl-, and phosphoryl-esters with a turnover. Albumin was shown to play a significant role in the detoxification of carbaryl at toxicologically relevant concentrations [44]. Thus, albumin in lymph and plasma may also scavenge certain OPs and play a role in their detoxification [45,46]. In addition, secondary OP targets present in various tissues participate in the neutralization of OP molecules. They also play a role in the natural protection of the cholinergic system [47]. However, as mentioned, the inactivation of certain secondary targets is responsible for the sub-lethal and chronic toxicity of OPs. Thus, despite this last issue, most of endogenous OP-scavenging and hydrolyzing enzymes and secondary targets can be regarded as the first-line of defense against acute OP poisoning [48].

## 3. Sources of Organophosphate-Degrading Enzymes (Fungal, Bacterial and Archaeal Sources, Engineered Enzymes)

Numerous studies have demonstrated the ability of micro-organisms to use OPs as a source of carbon (C), phosphorus (P), nitrogen (N), or sulfur (S). Other studies have proven that the degradation of OPs is possible through co-metabolism (the obligatory presence of a complementary substrate to provide the source of C and energy). In all cases, degradation is the result of the activity of enzymes secreted by the micro-organisms involved. Enzymatic catalysts capable of degrading OPs have been identified not only in microbial species, but also in eucaryotes like squid and mammals [49]. Some examples are shown in Table 1.

### 3.1. Organophosphate Degradation by Microbial Enzymes, Types of Enzymes and Mechanisms

The methods employed to isolate OP-degrading enzymes depend on their location in the microbial cells. They include cell-disrupting methods such as using silica or glass beads, ultrasonication, etc., to isolate enzymes located intracellularly, and cell centrifugation, filtration, etc. for those with extracellular locations.

**Figure 2:**
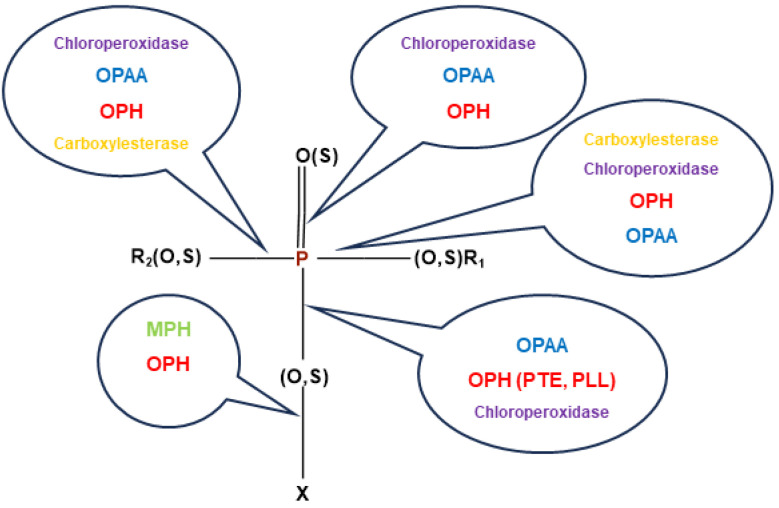
Different enzymes involved in the degradation of OP.

Multiple enzymes are involved in microbial hydrolytic degradation of OPs (Figure 2). However, bacterial cytochrome P450s (BacCYPs) dearylate aryl-containing OP groups (R_1_, R_2_) and play also a role in detoxification of these compounds.

#### 3.1.1. Phosphotriesterases

Phosphotriesterases (PTEs) are a group of enzymes that hydrolyze OPs. They are found in animals, micro-organisms, and plants. There are three different types of well-characterized PTEs: organophosphate hydrolase (OPH and OpdA), methyl parathion hydrolase (MPH), and organophosphorus acid anhydrolase (OPAA). The OP-degrading enzymes catalyze hydrolysis of either O-P, C-P, P-S, P-N or P-F bonds. OPs are broken down by enzymes through a nucleophilic attack on their phosphorus core. This attack is facilitated by two divalent metal ions, a water molecule, and reactive amino acids present in the enzyme’s active site [49,58].

OPH or PTE, also known as paraoxonase (PON), or aryl-dialkyl-phosphatase, was the first known enzyme (initially found in *Sphingobium fuliginis* (*Flavobacterium* sp.) and *Brevundimonas diminuta* (*Pseudomonas diminuta*)). This enzyme consists of two identical subunits with 336 amino acid residues. PONs can be found in various tissues in mammals, birds, fish, mollusks, and plants. However, it must be noted that bacterial and eucaryotic paraoxonases are structurally different and display different catalytic efficiency against OPs. These metalloenzymes are encoded by the OP-degrading (opd) gene [51,52,58,59,60]. Three different classes of organophosphorus hydrolase genes, namely, opd, mpd, and ophc2, were identified. The opd gene is widely distributed. The organophosphorus hydrolase genes opdA, opdB, opdC, opdD, and opdE were reported [61].

OPH effectively hydrolyzes organophosphate pesticides containing P-O, P-F, P-CN, and P-S bonds. Paraoxon, parathion, and diazinon are examples of OP insecticides containing P-O bonds that are efficiently hydrolyzed by OPH. It has been established that the optimal OP substrate for OPH is paraoxon [58]. Mutagenesis can be used to further enhance the various OPH enzymes’ capacity for hydrolyzing and detoxifying OPs [60].

Phosphotriesterases (PTEs and PLLs; EC 3.1.8.1) detoxify OPs [62,63]. PTEs have been isolated from numerous bacterial and archaea strains. These enzymes belong to a superfamily of amidohydrolases and determine four enzyme families of different folds: TIM-barrel fold, β-lactamase fold, pita bread fold, and β-propeller fold [64].

PTEs are promiscuous enzymes. Their primary function is lactonase. Thus, these enzymes are now called phosphotriesterase-like lactonases (PLL). The lactonase activity plays a role in bacterial communication (*quorum* sensing) [65,66]. Virulence and formation of biofilms are regulated by the concentration of lactones, the *quorum* sensing mediators, in the medium. Thus, the lactonase activity by hydrolyzing lactones acts as a *quorum* quencher, which in turn inhibits bacterial communication [67] and thus the formation of biofilms. The PTE activity is believed to have evolved from ancestral lactonases [68,69,70,71]. Recent reshaping of the active center conformation and plasticity of an archaea PPL supports this theory [72].

These enzymes are encoded by the organophosphate degradation (opd) gene present in *Brevundimonas diminuta* (formerly *Pseudomonas diminuta*), *Flavobacterium sp*., *Agrobacterium radiobacter* [73], and *Pseudomonas pseudoalcaligenes* [74]. Genes similar to opd were also found in archaeas [75].

Both OpdA and OPH belong to a broad family of enzymes that have a binuclear metal core and require two metal ions, such as Zn^2+^ (OpdA) or Co^2+^ (OPH), in the α and β sites for the hydrolytic reaction step. The coordinated two metal ions in OpdA engage with a hydroxide ion or water molecule as well as a carboxylated lysine residue (Lys169). Although 90% of the OPH identity is shared by the OpdA enzyme, there are some changes in substrate selectivity and kinetic behavior between them, according to homology studies. The most significant amino acid sequence differences between OpdA and OPH are as follows: (a) distinct residues in the active site, which are identified in OPH and OpdA as His254/Arg254, His257/Tyr257, and Leu272/Phe27, respectively; (b) 20 additional amino acids at OpdA C-terminus appear to be unimportant for catalysis because they are situated far away from the active site; (c) a complicated hydrogen bond network in OpdA allows two (Tyr257 and Arg254) of the three amino acid residues, close to the active site, to play a significant role in modifying the catalyzed reaction. In OPH, these hydrogen bonds are not as important [76].

**Figure 3:**
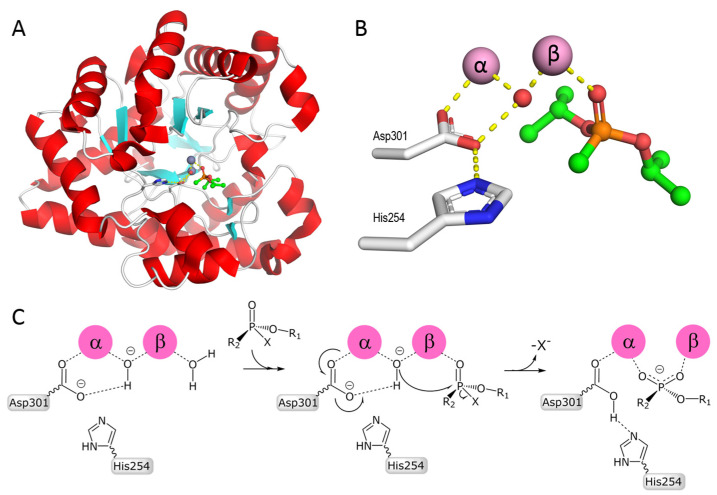
PTE from *Brevundimonas diminuta* (PDB ID 1EZ2) [86]. (**A**) overall view of crystal structure and (**B**) close view on bi-metal cation active center with bound substrate, diisopropyl methyl phosphonate. (**C**) Proposed hydrolysis mechanism scheme [87]. Reproduced with permission of Elsevier.

The most well-known enzyme is *Brevundimonas diminuta* PTE. It is a 72 kDa dimeric enzyme. Zn^2+^ is involved in the catalytic process [77]. The substitution of the native Zn cations in the active site with Mn, Co, Ni, or Cd cations results in almost full retention of catalytic activity. Following the first determination of the 3D structure of *Brevundimonas diminuta* PTE [78] (Figure 3A), a series of crystal structures, kinetic, and spectroscopic studies were reported. The oxygen atom seen in X-ray structures, coupled with two metal cations (Figure 3B), is thought to be in a hydroxyl form because the structure is pH-dependent and the protonation of hydroxyl leads to the loss of coupling [79]. The catalytic mechanism of PTEs is still debated and the functional roles of divalent cations and amino acids in the active center of these enzymes are not yet completely understood [64,80,81,82,83,84,85].

The catalytic mechanism proposed by Bigley and Raushel [64,87] for the *Brevundimonas diminuta* enzyme is the most accepted (Figure 3C). It states that PTE-catalyzed hydrolysis of OP results from a direct attack of the hydroxyl-group bridging divalent metal cations on the P atom. As a result, the formation of products is accompanied by the inversion of the phosphorus atom stereo-configuration. The hydrolysis product is bound to cations in a bidentate manner. Surrounding active center residues have a role in accepting proton from the hydroxyl-group upon formation of the negatively charged reaction product. Kinetic [79], crystallographic [88], electron paramagnetic resonance spectroscopy [83], NMR [89], and computational chemistry studies [84,90] support this mechanism. Lessons from eucaryotic PTEs, PON-1, and DFPase contributed to solve the puzzling mechanism of bacterial and archaeal PTEs. The catalytic mechanism of squid DFPase (DFP is diisopropyl fluorophosphate), a calcium-dependent PTE [64,91] was first proposed, involving a calcium-coordinated aspartate as the nucleophile pole to attack the phosphorus atom. However, a more realistic mechanism was proposed. In this mechanism a water molecule is activated, leading to a hydroxide ion prone to attack the phosphorus center [92]. This scheme is consistent with the general mechanism proposed for all PTEs: mammalian (PON-1) [93,94], PTEs [80], and PLLs [69,87] (Figure 3C).

Mechanisms of OP degradation by Opdh were also proposed. Mali et al. [53] studied the degradation of chloropyrifos by the opdh of *Arthrobacter* sp. HM01 and proposed two possible mechanisms. The first mechanism generates TCP (3,5,6-trichloro-2-pyridino) that will be successively transformed into DHP (2,6-di-hydroxy-pyridine), malic acid, and pyruvic acid. The latter product will be able to integrate the TCA (tri-carboxylic acid cycle). The second proposed mechanism generates DETP (di-ethyl-thio-phosphoric acid) that is subsequently transformed into phosphoric acid and will also integrate TCA.

OpdA, a variant of the OPH enzyme, is the only enzyme that is commercially used to bioremediate and clean up pesticide-contaminated water sources. OpdA is encoded by the opdA gene, obtained from *Agrobacterium radiobacter*. It can hydrolyze a wide range of OP pesticides [58]. Although the secondary structures of OpdA and OPH are similar, their active site structures are different, resulting in different substrate specificities. Typically, OpdA chooses substrates with fewer alkyl substituents. It may cleave substrates into phosphate ions and alcohols [49,51].

The catalytic efficiency (*k_cat_*/*K_m_*) of *Brevundimonas diminuta* PTE for hydrolysis of paraoxon, the model substrate, is approaching the diffusion-controlled limit (2 × 10^9^ M^−1^min^−1^ [95]). However, it is rather slow against malaoxon. Then, rational engineering of the enzyme allowed it to greatly improve its catalytic efficiency against malaoxon up to (*k_cat_*/*K_m_* = 4.6 × 10^5^ M^−1^ min^−1^) [96]. The catalytic activity of the wild-type enzyme is also slow against CWA (e.g., 6 × 10^5^ M^−1^min^−1^ against soman [97]). However, directed evolution of the enzyme showed that only three amino acids change dramatically and enhance the catalytic efficiency for an analog of soman by ~3 orders of magnitude [98]. Further studies combining rational design and directed evolution led to the selection of mutants from randomized libraries. The catalytic activity of these mutants against Sp enantiomers of nerve agent analogs and racemic real nerve agents was greatly improved [99,100]. This rational design approach led to multiple mutants with *k_cat_*/*K_m_* up to 4 orders of magnitude higher than that of wild-type PTE against V agents [87,101,102]. A study with the last designed mutants proved that in vivo detoxification of VX is possible [102]. A theoretical study suggested that enzymatic hydrolysis of novichok agents is also possible [103] and, indeed, a recent work showed that multiples mutants of *Brevundimonas diminuta* PTEs may degrade these OPs [104]. However, enzymatic fast hydrolysis of phosphoramidates like novichoks is a challenge, owing to electron delocalization along the P-bonded amidine chain, thus preventing the effective nucleophilic attack of water on the phosphorus atom.

Numerous studies highlight the potential of *Brevundimonas diminuta* PTE for surface decontamination and skin protection [62,105,106]. Administration of wild-type and mutants of this enzyme before or after OP exposure was shown to improve pharmacological pre-treatment and current treatment of OP intoxications [107]. However, in order to prevent abnormally fast pharmacokinetics and/or immunological response due to the injection of a bacterial enzyme, PTE could be PEGylated [108] or encapsulated. In vivo assays with PTE encapsulated in murine erythrocyte ghosts were promising [109]. Later, the encapsulation of PTE in liposomes provided protection of rats from multiple LD_50_ of paraoxon [110]. Blood detoxification through extracorporeal circulation devices, e.g., a cartridge containing immobilized PTE was proposed [111]. However, storage and implementation of such devices are difficult under field conditions [112]. Different formulations of PTEs were also evaluated for mild decontamination of mucous membranes and wounds as well as for skin protection in topical skin protectant creams or covalently coupled to the skin cornified layer [113]. However, long-term stability of these formulations impairs their practical use so far.

*Brevundimonas diminuta* PTE was also entrapped in additives and paints for surface coating. In particular, it was found to be effective in inhibiting quorum sensing and preventing formation of biofilms on different surfaces, including the hull of boats (https://www.gene-greentk.com) (accessed on 14 July 2024). PTE-containing additives were shown to retain the catalytic properties and stability of enzymes [114]. For the decontamination of the environment and remediation, phytodegradation of OPs by transgenic plants expressing a bacterial PTE has been considered as a potentially low-cost, effective, and friendly method [115]. Chemical modification of enzymes may improve their catalytic properties. For example, His-tagged PTE [116] was reported to degrade numerous OPs, including VX, at a high rate. Since the enzyme is the wild-type PTE, it is suggested that the presence of the His tag plays a role in this high activity. Though neither the 3D structure nor molecular dynamics of the modified enzyme are available, it can be hypothesized that the His tag may increase the enzyme flexibility, which in turn should improve the enzyme capability to accommodate OP molecules and increase its catalytic activity.

#### 3.1.2. Phosphotriesterase-like Lactonase (PLL)

PLLs are members of amidohydrolase family. Highly stable lactonases/phosphotriesterases (PLL) have been isolated from extremophilic environments like hot springs and volcano *solfatara* [117]. They are from hyperthermophilic archaeas (e.g., *Sulfolobus solfataricus*, *Sulfolobus islandicus*, and *Sulfolobus acidocaldarius*), hyperthermophilic bacteria, and aerobic bacteria such as *Pseudomonas pseudoalcaligenes*. PLLs have a (β/α) 8-barrel fold structure with a divalent metallic center, which governs the catalytic activity. They show PTE activity and hydrolyze lactones such as acylhomoserine lactones (AHLs).

SsoPox, isolated from *Sulfolobus solfataricus*, has a high potential for the degradation of OPs [118]. Mutants of SsoPox with significantly increased PTE activity were produced by genetic engineering. The SsoPox-αsD6 mutant is the most interesting of these mutated enzymes. Using an *E. coli* BL21(DE3)-pGro7/GroEL (TaKaRa, Shiga, Japan) chaperone-expressing strain, it was cloned into a pET32b-Δtrx plasmid and functionally expressed [55].

The 3D structures, evolution, stability and catalytic properties of several of these PLLs were determined [69,71,119,120,121,122]. These enzymes, wild-type and evolved mutants with improved catalytic efficiency (re-designed active center) against OPs, have been conveniently expressed in E.coli where their heat stability allows easy purification [71,120,123,124,125]. Owing to the high thermal stability of these archaea enzymes, allowing long-term storage at temperatures above room temperature, fieldable uses for different purposes are possible. Moreover, the techniques of encapsulation in nanoparticles, involving heat processes, do not cause denaturation of these enzymes during preparation of nano-formulations.

#### 3.1.3. Other Bacterial Enzymes Reacting with OPs

Other classes of hydrolases involved in the biodegradation of OPs have been discovered. Methyl parathion hydrolase (MPH) is a member of the β-lactamase superfamily. It is active against various OPs and is present in several phylogenetically distinct bacteria. However, its substrate range is smaller than that of OPH [126]. Each monomer in this homo-dimeric enzyme contains a hetero-binuclear Zn^2+^/Cd^2+.^ Zn^2+^ can be substituted by Co^2+^, Ni^2+^, and Mn^2+^, while Cd^2+^ can also be substituted by Co^2+^, Ni^2+^, Mn^2+^, and Fe^2+^. There is currently little knowledge about the MPH reaction mechanism. Three hydrophobic pockets comprise the active site of MPH [50]. Residues Leu65, Leu67, Phe119, Trp179, Phe196, Leu258, and Leu273 are part of the substrate binding pocket, while alanine substitutions of Phe196 and Leu273 enhanced enzymatic activity towards the substrate p-nitrophenyl diphenylphosphate, alanine substitutions of Phe119, Trp179, and Phe196 are detrimental to the catalytic activity towards methyl parathion [127].

Organophosphorus acid anhydrolase (OPAA) is also an important enzyme. With no structural or gene-sequence similarities to OPH or MPH (methyl parathion hydrolases), OPAA, encoded by the opaA gene, was shown to be a member of the dipeptidase family in *Alteromonas undina* and *Alteromonas haloplanktis* [58]. In 1992, the International Union of Biochemistry named the P-F or P-CN bond-degrading enzymes as OPAA. OPAA is a single-chain polypeptide with a molecular weight of 58 kDa, acting in a temperature range of 10–65 °C (optimal at 40–55 °C), and in a pH range of 6.5–9.5 (optimal at 7.5–8.5) [59].

This metalloenzyme is a tetramer (a dimer of a dimer) harboring binuclear Mn^2+^ ions in the active site. It can hydrolyze various OPs. In contrast to P-O or P-C bonds where the enzyme shows very little activity and P-S bonds that are resistant to hydrolysis, OPs with P-F bonds show a high degree of hydrolysis [19]. The active site is located in an oval pocket in the β-sheet part of the C-domain. There are three pockets at its binding site: small, large, and leaving. The small pocket is lined with Try212, Val342, and His343, and capped with Asp45 at the N-terminal domain of the opposing dimer subunit. Tyr292 and Leu366 are found in the leaving pocket, whereas Leu225, His226, His332, and Arg418 are linked to the large pocket, which is capped by Trp89 from a different subunit. According to the suggested mechanism for OPAA enzymes, two manganese (II) ions have a hydroxide bridge between them, which initiates a nucleophilic attack on the phosphorus center, resulting in the production of a transient intermediate that subsequently departs with the leaving group [118].

In addition, we must mention the potential interest of other extremophile OP-reacting enzymes isolated from halophilic bacteria (*Alteromonas*), such as the OPAA (organophosphorus acid anhydrolase), and from radio-resistant bacteria, *Deinococcus radiodurans* and *Agrobacterium radiobacter*. The 3D structure and catalytic mechanism of these enzymes were also determined and used for a structure-based random mutagenesis rational design to improve enzyme catalytic efficiency against OPs [128,129,130]. Recent mutagenesis of OPAA generated new mutants against chemical warfare nerve agents (CWNAs); one of these mutants displayed the highest activity against soman [131]. Mining in genomic databases also allowed the discovery of a new OP scavenging enzyme: esterase-2 from the planctomycetota hyperthermophilic bacterium *Thermogutta terrifontis* [132]. Although this enzyme is not very effective, its catalytic activity could be improved, and it also demonstrates that mining research in databases is promising. Moreover, new and fast fluorimetric screening methods allow the identification of highly active PTEs in micro-organisms from various biotopes [133].

Bacterial and archaea prolidases are also promising enzymes [134]. Prolidases (EC 3.4.13.9, PROL) were first isolated from halophilic bacteria (*Alteromonas haloplanktis* and A. sp. JD6.5). This metallo-enzyme (binuclear Mn^2+^ center) has a “pita bread” structure [135]. Prolidase from A. sp. JD6.5 is an OPAA that displays a high activity against soman (*k_cat_* = 3100 s^−1^ with *k_cat_*/*K_m_* = 1 × 10^7^ M^−1^min^−1^), but it is inactive against VX [136,137]. Thermostable prolidases from hyperthermophilic archaeas *Pyrococcus furiosus* [138] and *Pyrococcus horikoshii* [139] hydrolyze P-F and P-O bonds in nerve agents. Evolved mutants of these enzymes capable of degrading OPs over a wide temperature range, like engineered hyperthermophilic PLLs, should have a future for enzymatic bio-decontamination.

Bacterial ChEs have been known for long time [140]. A 43 kDa AChE-like enzyme from *Pseudomonas fluorescens* was isolated [141]. This enzyme displays a low sensitivity to OPs with a bimolecular rate constant of the order of 0.5 × 10^2^ M^−1^.min^−1^ with echothiophate and DFP (diisopropylfluorophosphate). The phosphorylated enzyme cannot be reactivated by oximes. More recently the 3D structure of a related AChE-like from *Brevundimonas diminuta* was solved [142]. This 30 kDa enzyme has a α/β/α fold distinct from the α/β fold of eucaryotic ChEs and displays low sequence homologies with other ChEs. However, its catalytic triad resembles that of ChEs. Despite differences with eucaryotic ChEs, the functional convergence between eucaryotic and procaryotic ChEs could be exploited. In particular, knowledge of the 3D structure and molecular dynamics of AChE and BChE [143] opened a way to rational re-design of ChEs to OP hydrolases. In particular, the possibility to convert ChEs into an OP hydrolase (OPH) has been attempted [144]. However, mutated enzymes display low OPase activity and the mechanism of dephosphylation of these mutants is still debated [145]. Nevertheless, the computer-assisted design of new mutants of ChEs is conceivable. “Intelligent” directed mutagenesis design based on the simulation of reaction mechanisms, modeling of intermediates and transition state structures with quantum mechanics (QM) and quantum mechanics/molecular mechanics (QM/MM) calculations along dephosphylation reaction coordinate may allow the design of highly active mutants. Thus, new molecular dynamic methods, using principal component analysis and Markov chain models could be implemented to explore reaction paths before construction of designed mutants. Application of these methods to bacterial ChEs is thought to speed up the process of mutant creation and to considerably decrease the cost of their functional expression.

Bacterial CaEs can break down malathion by cleaving one or two carboxyl groups to produce mono- or di-acid derivatives, but OP-reacting properties of these enzymes have not been extensively explored. Also, fungal cutinase, a lipolytic enzyme exhibiting a high initial malathion degradation rate (approximately 60% in 30 min), can generate malathion monoacid and malathion diacid. Yeast esterase, a lipolytic enzyme obtained from *Lysinibacillus* sp. KB1, can degrade malathion and generates malathion dicarboxylic acid and malathion monocarboxylic acid [60].

Oxidases are also of interest, in particular laccases. Laccases (EC 1.10.3.2) are phenol oxidoreductases. Laccase from *Pseudomonas sp*. S2 produced in a bioreactor was found to oxidize OP pesticides in a short time [146]. Moreover, phosphorothiolates (P-S bonded OPs) and phosphoramidates (P-N bonded OPs) are almost resistant to PTEs. Thus, the oxidative cleavage of the P-S and P-N bonds could be achieved by oxidases like laccases. These enzymes could be used in medical countermeasures in association with other OP-degrading enzymes. Though no work has been reported on the combined action of oxidases and hydrolases, the oxidation of P-bonded alkyl/aryl chains by oxidases is expected to alter the enantioselectivity of PTE for parent OPs. Therefore, biopharmaceutical formulations in which oxidases and PTEs are combined may improve the efficiency of catalytic bioscavengers. Nevertheless, bacterial laccases could at least be used for decontamination and environment remediation [147]. Moreover, enzymes that degrade OPs are of interest for the destruction of CW stockpiles, decontamination of materials and protective equipment, and water polluted by pesticides and CW OPs [148].

Chloroperoxidase, a fungal peroxidase from *Caldariomyces fumago* is capable of converting OP insecticides that have phosphorothioate group (P=S). However, the oxidized products were found to be oxon (P=O) derivatives, in which an oxygen atom has taken the place of the sulfur atom from the thioate group. These oxon forms are more hazardous than the parent insecticide [57]. There are also several other enzymes, such as aldehyde oxidase, esterase, glutathione S-transferase, reductive dehalogenase, dioxygenase, aminopeptidase, nitroreductase, laccase, and peroxidase, that are produced by several microbes and have been reported to degrade a variety of OP pesticides [149]. However, like chloroperoxidase and cytochromes P450, they may lead to more toxic OPs.

Protein engineering has made extensive use of these techniques as a standard method for improving protein function by chemical modification or protein fusion. For instance, it was interesting to discover that, while hydrolyzing parathion and methyl parathion, an N-terminal dodecahistidine tag (His12-OPH) increased catalytic efficiency by 30 and 74 times, respectively, compared to wild-type OPH. It was also intriguing to learn that by lengthening the polyhistidine tag from six to twelve His residues, the ideal pH of the fused OPH could be progressively moved to the alkaline range. Furthermore, decreased thermostability at temperatures below 50 °C and increased thermostability at temperatures above 50 °C were caused by the tendency of His12-OPH to oligomerize. OPH fusion with alternating glutamic acid and lysine sequences (EK) of 30 kDa at the C-terminus was another intriguing occurrence. When compared to wild-type OPH, the fusion disrupts the formation of OPH dimer and produces a stable monomeric OPH that exhibited a modest increase in thermostability and a 70% increase in substrate affinity by reducing *K_m_* [50].

Research, isolation, and engineering of microbial enzymes capable of neutralizing OP either as stoichiometric or catalytic bioscavengers have been undertaken in collections of known micro-organisms, natural environments polluted by OPs, extreme biotopes, and mining in protein databases DNA sequences [132,150]. Finally, mining of these enzymes and DNA sequences of interest, mutagenesis and functional expression in simple bacterial hosts (e.g., *E coli*), and alternatively, engineering (computer design and/or directed evolution) of known enzymes capable of degrading OPs are the most promising short-term research fields to obtain effective enzymes of interest.

## 4. The Medical Bioscavenger Concept

**Figure 4:**
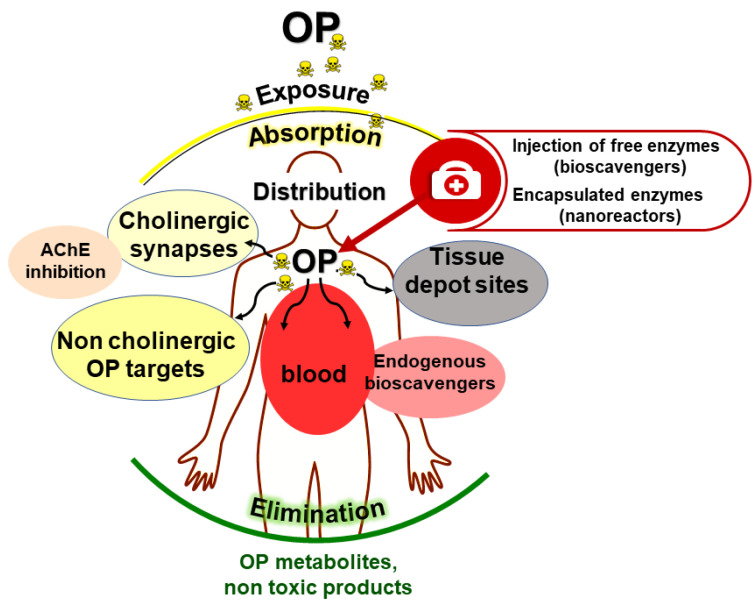
Biological fate of organophosphorus compounds in poisoned organisms. Routes of penetration of OPs are absorption through the skin, eyes, and/or respiratory tract (nerve agents and pesticides), or ingestion (self-poisoning). OP molecules are distributed from the blood compartment into tissues, including depot sites, physiological targets, and sites of elimination (liver and kidneys). ChEs are the main targets (see Figure 1). Reaction of OPs with secondary targets (carboxylesterases, serine-amidases, peptidases, and other proteins) may be responsible for non-cholinergic sub-lethal effects of OPs and chronic toxicity at low dose exposure. (Adapted from [112]).

Lessons from endogenous OP-reacting enzymes and proteins show that the acute toxicity of OPs can be countered by dramatically lowering OP concentrations in the blood compartment. This can be achieved by trapping/inactivating OP molecules on the skin and exposed mucus membranes, and in the bloodstream. The neutralization of OP molecules prevents their transfer to cholinergic synapses (peripheral cholinergic system nodes, central nervous system and neuromuscular junctions) and other biological targets (Figure 4).

The concept of bioscavenger and developments of this medical approach in prophylaxis and post-exposure treatment of OP poisoning have been covered in several reviews [151,152,153,154].

The use of bioscavengers is the most effective alternative approach for the neutralization or detoxification of OPs and surface decontamination under mild conditions, pretreatment or prophylaxis, and post-exposure treatment of OP poisoning. The administration of bioscavengers by i.v or i.m leads to the neutralization of toxic molecules in the bloodstream before they reach physiological targets, thus providing protection against poisoning.

First-generation bioscavengers are stoichiometric enzymes that react mole-to-mole with OPs. However, considering the molecular mass ratio bioscavenger/OP, stoichiometric neutralization of OPs needs the administration of huge amounts of costly biopharmaceuticals [155]. The second generation of bioscavengers or catalytic bioscavengers are enzymes using OP as substrates. They neutralize OPs with a turnover and therefore need to be administered at much lower doses than stoichiometric bioscavengers for the same efficacy [156]. Catalytic bioscavengers could also be introduced in protective topical creams. Thus, the introduction of catalytic bioscavengers in protective devices and as medical counter-measures against OP poisoning considerably improves the efficacy of prophylaxis and post-exposure treatments.

### 4.1. Stoichiometric Bioscavengers

Starting from the end of the 1980s, research on bioscavengers mostly focused on enzymes that specifically react with OPs, in particular human BChE. Later, human BChE has proved to be an effective stoichiometric bioscavenger for pre- and post-exposure treatment of OP poisoning by pesticides and CWA [157,158,159]. Among secondary targets of OPs, albumin is certainly one of most interesting proteins. Owing to the high number of albumin residues that covalently bind OPs (5 tyrosines and 2 serines) [160], it may be hypothesized that the reactivity of these residues could be enhanced by genetic engineering and/or upon specific chemical modification. New reacting residues could also be made by site-directed mutagenesis. Thus, engineered albumins is thought to lead to novel stoichiometric bioscavengers. However, the conversion of albumin into a catalytic bioscavenger is presently unrealistic because this would imply the increase of the catalytic efficiency (*k_cat_*/*K_m_*) by several orders of magnitude.

The main limitation of stoichiometric bioscavenger is the cost because huge doses of enzymes have to be administered for challenging the OP molecules [155] without inducing unwanted side reactions. A way to circumvent the dose limitation is to in reactivate the administered enzyme in vivo after reaction with OP molecules, turning the stoichiometric bioscavenger into a pseudo-catalytic bioscavenger.

### 4.2. Pseudocatalytic Bioscavengers

Because OPs phosphylate the active site serine of ChEs (Figure 1, reaction 2), OPs may be regarded as pseudo-substrates of ChEs [2]. When ChEs react with substrates, i.e., carboxyl-esters, there is a rapid turnover: after formation of the Michaelian complex, the acyl-enzyme intermediate is transiently formed, and then the acyl group is rapidly displaced by a water molecule acting as a co-substrate. On the contrary, in the case of OPs, because of the stereochemistry of the phosphyl-enzyme intermediate (Figure 1, reaction 2), the accessibility of water for attacking the phosphorus atom is restricted, and the enzyme remains phosphylated, i.e., irreversibly inhibited. However, certain ChE mutants not susceptible to age after phosphylation can be reactivated by nucleophilic agents (Figure 1, reaction 3). For instance, the human AChE double mutant Y337A/F338A [161] in the presence of oxime is reactivated and acts as a pseudo-catalyst in displacing the OP group bound to the active site serine. Such a mutated enzyme coupled to a reactivator could behave like a pseudo-catalytic bioscavenger [162]. A first practical realization of such a self-reactivating system was reported by [163]. The authors made a polymer-oxime-BChE macro-conjugate capable of reacting with OPs with the subsequent slow self-reactivation of the enzyme due to the associated multiple oxime moieties.

However, the practical efficiency of pseudo-catalytic bioscavengers in vivo requires the implementation of new oximes, displaying higher affinity for phosphylated ChEs, higher reactivation constant (*k*_r_), and long residence time in the bloodstream. Moreover, the pharmacokinetic profiles of both enzymes and reactivators must be similar. The enzymes can be chemically modified for long residence times in the bloodstream. However, the clearance of oximes in blood is, in general, fast. To circumvent pharmacokinetic issues, oximes can be either encapsulated into nanocontainers for slow release, and thus, prolonged action in the bloodstream [112] or both enzyme and oximes can be co-encapsulated into the circulating enzyme nanoreactors, where coupled reactions of bioscavenger phosphylation and subsequent oxime-mediated reactivation of phosphylated enzymes take place [164].

### 4.3. Catalytic Bioscavengers

Catalytic bioscavengers are enzymes or catalytic antibodies capable of degrading OPs with a turnover (*k_cat_*). These catalysts detoxify OPs by hydrolyzing phosphoester bonds. Organophosphorus acid anhydride hydrolases (OAAH), OP hydrolases (OPH, OPase), phosphotriesterases (PTE) and prolidases that catalytically hydrolyze OPs, as we said, can be used as catalytic bioscavengers. Other enzymes, like oxidases, lead to less toxic compounds by degrading their alkyl/aryl chains through oxidation. Several reviews deal with catalytic bioscavengers, in particular those of bacterial origin [87,165,166,167]. Thus, the catalytic bioscavenger concept is based on the idea of continuous degradation of OP substrates with a turnover after the administration of these enzymes. As for stoichiometric bioscavengers, these enzymes act in the bloodstream and neutralize OPs before toxic molecules reach physiological targets. Then, prophylactic injection of enzymes capable of hydrolyzing OP quickly (alone or in association with current prophylactic countermeasures), would allow workers and specialized personnels to operate safely in contaminated environments or to provide medical assistance to contaminated casualties under safe conditions. Administration of catalytic bioscavengers to poisoned casualties is also expected to greatly improve the efficacy of classical pharmacological countermeasures [168,169,170]. In addition, catalytic bioscavenger formulations could be implemented for skin protection in nano-formulations [171], and in decontaminating solutions for body decontamination [105,106]. For examples, a few practical enzyme formulations for OP decontamination have been marketed so far, e.g., VesuTOX (www.gene-greentk.com). Genetically engineered bacteria producing OP hydrolases can also be used for decontamination of water effluents as well as for purification of contaminated water before recycling or washing up in the environment [172]. At this point, medical applications of catalytic bioscavengers merge with environmental applications for remediation.

## 5. Requirements for Efficacy of Procaryotic Enzymes to Detoxify OPs

### 5.1. Requirements in Medicine for Efficacy and Safety of Injected Bacterial Enzymes

The general requirements for the medical uses of OPs-degrading enzymes against OP poisoning are as follows: (1) enzymes must effectively react with a broad spectrum of OP molecules (the association of cocktails of several enzymes, displaying different specificity towards OPs also enlarges the spectrum and may lead to multiple enzyme formulations toward several toxicants and for multipurpose uses) and ideally, these enzymes must display enantioselectivity for the most toxic OP stereoisomers; (2) the enzymes must not induce iatrogenic effects after injection or in topical application; (3) mass production of highly purified, free of detectable contaminant, sterile wild-type and mutant enzymes under good manufacturing practice conditions must be realizable at reasonable cost. For instance, the cost of one dose (200 mg) of the first generation bioscavenger, human plasma-derived BChE was estimated to be higher than USD 20,000. This high cost considerably limited the practical interest of stoichiometric bioscavengers for medical treatments. In the case of catalytic bioscavengers of microbial origin, the injection of much lower doses of enzymes (e.g., <10 mg) with high catalytic efficiency (*k_cat_*/*K_m_* > 10^6^ M^−1^ min^−1^) leads to much lower costs. The acceptable cost must be about USD 50 per dose); (4) long storage (several years) without activity loss (either in lyophilized form, in solution or adsorbed/bound on a matrix, in a gel or in a foam) must be feasible. This issue is mandatory, owing to the cost of enzyme production. The conformational stability of enzymes is an issue, in particular for storage and field uses. It can be increased by chemical modifications or the addition of stabilizers likes polyols, e.g., threalose. Otherwise, the use of thermostable PLL-PTEs from hyperthermophilic bacteria/archaea [69,75] expressed in *E. coli* or mutated/evolved highly stable enzymes from mesophilic bacteria are alternatives.

**Figure 5:**
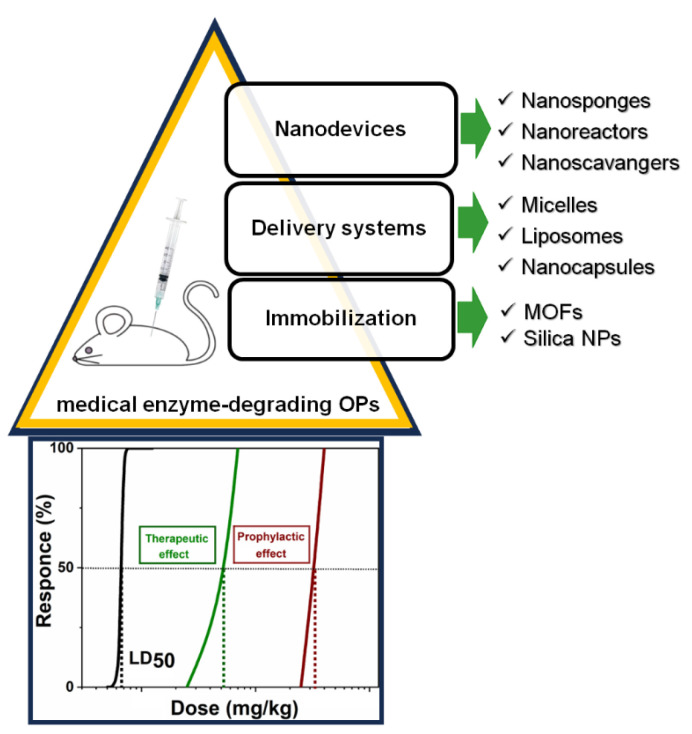
Nano-biotechnological strategies for implementing bacterial/archaeal enzyme-degrading OPs for medical purposes. The dose–response plot shows the prophylactic and post-exposure treatment efficacy of a PTE-nanoreactor in mice challenged with paraoxon [198,199].

Currently, several nanotechnological strategies are known for implementing bacterial OP-degrading enzymes for a medical purpose (Figure 5). Immobilization strategies, involving adsorption, covalent binding or copolymerization, and preparation of nanocomposites, Metal−Organic Frameworks (MOFs) [173,174], and silica nanoparticles [175] are the first solution. At the same time, traditional delivery systems for encapsulating bacterial enzymes such as recombinant PTEs, organophosphorus hydrolase using red blood cells [176], sterically stabilized liposomes [177,178,179,180], poly(2-ethyloxazoline)-based core shell dendritic polymer micelles [181], capsules [175,182], and nano-complexes [183] as vehicles can be developed. In the case of immobilization and encapsulation, there is a decrease in effectiveness of injected enzymes due to the diffusion through membrane or pores of encapsulating material and rapid clearance by the immune system. These solutions have other limitations for biomedical applications; one of the main concerns is biosafety (biocompatibility, iatrogenicity and long-term toxicity, immunogenicity, and pharmacokinetic issues (distribution, absorption, excretion and metabolism) [184,185]. There are few examples in the literature where the catalytic efficiency of immobilized enzymes is maintained or increased compared to the efficiency of free enzymes in buffers [173,186]. Therefore, recently, to improve the effectiveness of nano-therapeutic drugs, a new alternative approach was developed: the creation of biomedical robotic nanodevices for detoxification. Unlike traditional passive nanotherapeutics, nanodevices can perform various complex biomedical functions in the event of unexpected biological events [187,188]. Typically, traditional drug delivery systems aim to encapsulate therapeutic agents and release them into target tissues under the control of external stimuli. In contrast, nano-detoxifying devices, which are one or multicompartment devices with a size close to 100 nm, remove drugs and xenobiotics from biological tissues [189,190]. Optimizing physicochemical parameters such as the size and ratio of functional components, biocompatible broad-spectrum polymer nano-antidotes can facilitate rapid advancement into clinical uses [191]. Among them we must mention nano-sponges [192,193,194], nano-scavengers [195,196,197], and nanoreactors [198,199]. Usually, nanoreactors are two-phase systems [200] such as vesicles, polymersomes, proteinsomes, and capsosomes. They found application as mimics of organelles and living cells [201]. The large surface area of these nano-compartments promotes faster reaction rates compared to bulk materials with immobilized enzymes. The probability and efficiency of reaction increase due to the spatial limitation of reaction mechanisms and reagents getting inside and interacting with encapsulated enzyme(s). In addition, biocatalytic reactions can proceed with higher selectivity or fewer side reactions in a confined space.

The use of enzymes in effective enzyme nanoreactors for prophylaxis and post-exposure treatment of paraoxon poisoning illustrates the interest for such encapsulated enzyme systems: it significantly reduces mortality and intoxication symptoms [198,199], improves tolerance to poison and attenuates oxidative stress and organ damage [196,202], and penetrates the BBB to eliminate intracerebral OP molecules, thus impairing/limiting oxidative stress, neuroinflammation, and neuronal apoptosis of neurocytes [203]. This thereby demonstrates the unique functionality of these biomedical nanodevices.

Specific efficacy requirements depend on the way of administration, delivery system, or pharmaceutical formulation. Enzymes can be injected intravenously, intramuscularly, subcutaneously, or administered via the intranasal way. For the optimal efficacy of administered enzymes, acting as catalytic bioscavengers, knowledge of the toxicant concentration profile versus time in blood, i.e., its toxicokinetics is very useful for optimizing therapeutics. In most cases, it is difficult to determine. Otherwise, the determination of [*OP*]*t*, at fixed times after *t*_0_ (*t*_0_, exposure time) and determination of its irreversible inhibitory action on a reporter enzyme, e.g., BChE, must be considered [204]. It should be noted that even in the most severe case of poisoning, [*OP*] is always very low. For example, the concentrations of sarin in serum of casualties after the Matsumoto and Tokyo chemical attacks in 1994 and 1995 were estimated between 1.5 and 30 nM, 14 h after poisoning [205]. Thus, in human plasma, [*OP*] << *K_m_* of catalytically competent enzymes react with OPs as substrates. Therefore, under such reaction conditions, the kinetics for enzymatic hydrolysis of OPs in blood is always first-order [111,156] so that the simple Michaelis–Menten rate (*v*) equation reduces to Equation (1):

(1) $$v=k_{cat}/K_{m}\cdot \left[E\right]\left[OP\right]$$

In this equation, the product of the bimolecular rate constant (*k_cat_*/*K_m_*) and the enzyme active site molar concentration ([*E*]) is the first-order rate constant (expressed in min^−1^). Therefore, the enzyme dose to be injected for degradation of the toxicant in a very short time depends on the enzyme catalytic efficiency, i.e., *k_cat_*/*K_m_*. The higher this parameter, the lower the enzyme dose to be administered. The enzyme concentration needed to drop the *OP* concentration to a non-toxic concentration in time *t*, as short as possible, is:

(2) $$\left[E\right]=\frac{X}{k_{cat}/K_{m}\cdot t}$$

In Equation (2), *X* is the factor by which [OP] is reduced in time *t* (*X* = Ln([*OP*]_0_/[*OP*]*_t_*). Owing to the fast flow rate of blood circulation in human, with the average time of 1 min for a complete cycle, *X* must be estimated per minute. The *X* value must be high to prevent transfer of highly toxic OP molecules from blood compartment to nervous system targets. Because the cost of enzymes is still a limiting factor, [*E*] cannot be dramatically increased. Thus, this is the catalytic efficiency that must be optimized. As written above, *k_cat_*/*K_m_* and the stereospecificity of enzymes can be increased by several orders of magnitude by site-directed mutagenesis, directed evolution, or chemical engineering [98,100,167,206,207]. Engineering strategies to increase *k_cat_*/*K_m_* have been theorized [208]. The implementation of artificial intelligence algorithms is expected to soon lead to potent computer-designed enzyme mutants.

In the case of enzyme nanoreactors, where kinetics of degradation take place under second order conditions ([*E*] > [*OP*]), *k_cat_*/*K_m_* has also to be as high as possible [164] for inactivation in a very short time. Moreover, in Equation (2), it is assumed that the operational stability of the administered enzyme has been optimized so that [*E*] in the bloodstream must not decrease too rapidly during the time course of the reaction with OP molecules in blood. In fact, [*E*] must be maintained as high as possible for a long time. [*E*] is controlled by pharmacokinetics/pharmacodynamics and the frequency of repeated administrations of the enzyme preparation (sustained pharmacokinetics). Increasing the size of the enzyme by polymerization, conjugation to other proteins (e.g., albumin, antibody fragments) or to biodegradable polymers, and chemical modifications (“capping” of solvent-exposed surface) improve the operational stability, i.e., the residence time in blood of injected enzymes. It must be noted that fast clearance of bacterial enzymes may result from their small size [209]. Enzyme clearance can be slowed down either by chemical modifications such as PEGylation, polysialylation, and other conjugations, e.g., to dextran or other macromolecules, including proteins like albumin. Also, as for other detoxifying enzymes [210], nanoencapsulation of enzymes into nanocarriers (“nanoscavengers”) may greatly increase their residence time in the bloodstream [195] and suppress potential adverse effects such as immuno-reactivity. However, the possible partial encapsulation of large molecules such as enzymes in nanocontainers, thus forming a “corona”, may impair the advantage of nanoencapsulation.

**Figure 6:**
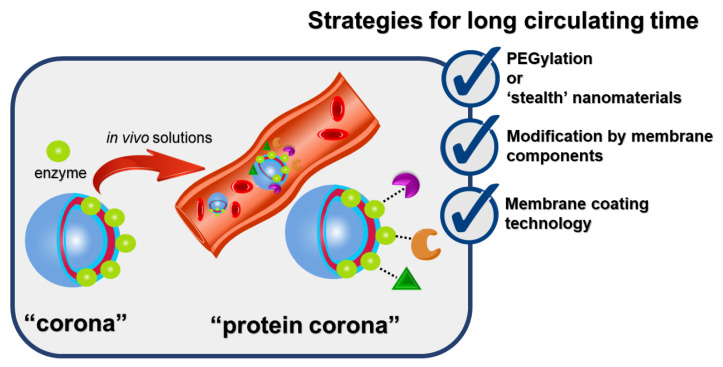
Nanotechnological strategies to avoid fast clearance and prolong the circulation of enzyme-loaded nanoparticles in the body.

Catalytic properties of membrane-bound or membrane-anchored enzymes can deteriorate [211,212] and depend on curvature, molecular density, packing defects, and thickness of membrane [213]. Enzymatic activity is associated with the availability of substrate for the enzyme located in the membrane and will be maintained in the case of a favorable orientation of enzyme on the surface of nanostructures [214]. Enzymes embedded within the nanoreactors or surface localization will complicate the formation of a protein corona on surface of nanoparticles (Figure 6).

The “protein corona” is a natural protein layer that spontaneously forms around nanomaterials in biological environments due to interaction with proteins, lipids, and sugars, acquiring new physicochemical properties. The formation of a protein corona changes the biological characteristics of nanoparticles, such as accumulation in tissues, cellular uptake, clearance by the immune system, and toxicity. It is extremely important to identify the type of proteins adsorbed on the surface of nanoparticles. Because, depending on their type, these proteins can either shorten or lengthen the circulation time of nanoparticles in the body [215]. Therefore, characterization of this protein layer will be a decisive step in the development of new nanomedicines [216]. At the same time, the understanding of the correlation between the physicochemical characteristics of nanoparticles and protein adsorption is improving [217]. One of the first strategies is to reduce or prevent the formation of the protein corona using stealth systems. One of the most common surface modifications is PEGylation. Such systems also include polyvinylpyrrolidone, peptides, and carbohydrates. It is impossible to completely prevent protein corona, so the next strategy is modification with membrane components and to attach NP with specific ligands or biomolecules: antibodies, protein fragments, peptides, or membrane protein CD47 [218]. It is important to note that the phenomenon of protein corona formation becomes more complex in the case of protein-surface or protein–protein interactions [219]. Moreover, weak interactions stabilize NPs, and strong protein–protein interactions cause NP aggregation [220]. The third step is the creation of biomimetic nanostructures or membrane coating technology of nanoparticles with plasma membranes of various cell types, e.g., erythrocyte, macrophage, and cancer cell membranes. The use of red blood cell-camouflaged nanoparticles increases circulation time and changes the pharmacokinetic profile and premature elimination from the body [221]. Synthetic membrane engineering described for more than 2 × 10^6^ BChE molecules bound to erythrocyte and provided the first line of defense against OP nerve agents exposure [222]. Another example showed the cell membrane acting as an emulsifier to stabilize a nano-sized oil droplet during emulsification and simultaneously increased detoxification efficiency and trapping of OP molecules [202]. Dual coating of MOF nanoparticles containing recombinant organophosphorus hydrolase with liposomal-lipid and erythrocyte membranes ensured not only the survival of mice after OP poisoning, but also biocompatibility, prolonged pharmacokinetics, and overcoming of the BBB [196,203]. Despite the exciting literary results, comparison between these studies are still challenging, mainly due to the lack of standardization of the analysis of protein corona [223].

The administration of homologous enzymes does not induce immune response following a second injection [224]. On the other hand, immunotolerance of injected heterologous enzymes is a major issue. Bacterial and archaeal enzymes may not be suitable for use in humans, but conjugation to dextran or polyethylene glycol may be sufficient to reduce antigenicity, non-specific immune response, and to slow down clearance following multiple injections [225,226]. Nanoencapsulation of non-human enzymes allow it to cheat the immune system and to increase the residence time of enzymes in the bloodstream [195,210]. However, enzyme-containing nanocontainers must be completely sealed to prevent leaks. This implies sophisticated design of decorated and crosslinked multilayer nanoparticles. Multicompartment structure allows the retention of therapeutic proteins and peptides for longer time. There are many techniques to create such structures. For example, multivesicular liposome (DepoFoam) technology can be used to develop prolonging therapeutic treatments and to reduce administration frequency [227,228]. Multiple emulsions can be fabricated, using microfluidic devices [229]. Polyelectrolyte multilayer nanoreactors and layer-by-layer (LbL) self-assemblies are found in the literature [230]. Although the biological activity of molecules is retained [231] nonetheless, the integrating proteins and enzymes in LbL thin films is still challenging for nanomedicine aims [232]. An alternative to the injection of enzymes is to incorporate OP-degrading enzymes in medical dialysis systems. This approach could greatly improve the efficiency of hemodialysis. In this respect, OP-reacting enzymes can be immobilized on dialysis cartridge membranes [233]. Co-immobilization of different enzymes could be an easy way to extend the spectrum of OPs to be degraded. Accessibility of OP molecules to the enzyme active center must not be altered by the immobilization method or by matrix effects. [E]/surface has to be maximized to reduce diffusion constraints and increase the reactive surface. Again, *k_cat_*/*K_m_* has to be as high as possible and the flow rate must be reduced to increase the efficiency of the detoxification process. First-order degradation kinetics takes place under the particular conditions of immobilized enzymes in the continuous-flow system. The above-mentioned enzyme nanoreactor approach could be an alternative to extracorporeal immobilized enzyme-cartridges [112]. Lastly, in situ transient production of enzymes should be possible by gene therapy in the future.

For external uses, in decontaminant solutions and topical protectants, enzymes have to be highly concentrated and display high catalytic activity. The situation is similar to that of enzyme nanoreactors, where the concentration of encapsulated enzyme is high. In fact, enzyme nanoreactors could be embedded in creams or gels for making active topical skin protectants. A mixture of different enzymes should allow simultaneous detoxification of various OPs and other types of toxicants. In particular, exposure to multiple toxicants cannot be ruled out in worse chemical warfare scenarios. Lessons from previous conflicts and terrorist acts remind us.

### 5.2. Requirements for Environmental Applications

Presently, there is an increasing interest in the use of enzymes in pesticide bioremediation. By using enzymes for OP degradation, it is possible to develop more effective and sustainable methods for detoxifying contaminated environments. Additionally, enzyme engineering techniques can improve the activity and stability of these enzymes, increasing their effectiveness in degrading OPs. The use of OPs-degrading enzymes has been the focus of research since the last two decades or so.

Haque et al. [234] observed the degradation of nine OP insecticides (ethoprophos (EP), parathion (PT), chlorpyrifos (CP), dyfonate (DF), cadusafos (CS), coumaphos (CM), methylparathion (MPT), diazinon (DZ), and fenamiphos (FA)) at 100 mg/L by two organophosphorus hydrolase A and E (OpdA and OpdE) from *Leuconostoc mesenteroides* WCP307. The results indicated that both enzymes were capable of differentially degrading the OP insecticides. The two OP hydrolases were very effective in eliminating PT, CP, DZ, MPT, and CM with 86, 79, 77, 74, and 71% degradation efficiencies, respectively, for OpdA, and 82% (PT), 77% (MPT), 75% (CP), 75% (CM), and 71% (DZ) for OpdE. The two enzymes showed optimal activities at 30 °C and at pH of 7.0 and 6.0, respectively. Both enzymes displayed optimal activities at 30 °C but under different pH conditions of 7.0 (OpdA) and 6.0 (OpdE), respectively.

Fang et al. [13] demonstrated the degradation of the insecticide profenofos (10 mg/L) with the help of organophosphorus hydrolase (OpdB) from *Cupriavidus nantongensis* X1T and expressed in *Escherichia coli* BL21 (DE3), using the expression vector pET22b-opdB. The tested enzyme showed optimal degradation activities of 46% at 37 °C and 50.6% at neutral pH. Also, it was observed that divalent metal cations (Ni^2+^, Mg^2+^, Co^2+^, and Ca^2+^) can increase the enzyme degrading activity, contrary to trivalent metal cations (Fe^3+^ and Cr^3+^), acting as strong inhibitors of the enzyme.

The degradation of methyl paraoxon (2 mM) was studied using OP hydrolases (Opds) from four fungal species, namely *Penicillium nalgiovense*, *Fusarium* sp., *Aspergillus niger*, and *Penicillium chrysogenum* [235]. The concentrated enzyme extracts from the fungal strains displayed methyl paraoxon degradation rates between about 38 and 80% after a 30-day reaction period in an acidic environment. The Opd from *Penicillium chrysogenum* with the best degradation performance (80%) exhibited optimal activity at 30 °C and strong stability, retaining 80% of its initial activity after 12 h when assayed with 22.5 µM methyl paraoxon under acidic conditions (pH 2) and in the presence of detergent (9.6% SDS). Moreover, the application of the enzyme in the bioremediation of apples contaminated with 8.5 mg/kg of methyl paraoxon gave a higher catalytic rate (6.2 nM/min at 30 °C, pH 2, and 9.6% SDS) when compared with the use of the genetically modified SsoxPox enzyme (5 nM/min at 25 °C and pH 7).

In another study, PTEs from six bacterial strains (*Arthrobacter oxydans* ATCC 14358, *Arthrobacter oxydans* ATCC 14359, *Nocardia asteroids* ATCC 19296, *Nocardia corynebacterioides* ATCC 14898, *Streptomyces setonii* ATCC 39116, and *Streptomyces phaeochromogenes* CCRC 10811) were tested for their ability to break down different OPs (coroxon, paraoxon, methyl paraoxon, chlorpyrifos, methyl parathion, coumaphos, and dichlorvos). The PTE extracts from each bacterial strain were assayed separately in the presence of 0.15 mM of each OP for 21 days. Generally, enzyme extracts exhibited greater PTE activity compared to the entire cells. Interestingly, PTE from *Arthrobacter oxydans* ATCC 14359 achieved complete degradation of methyl parathion, and 80% and 82% paraoxon and coroxon hydrolysis, respectively, were recorded with PTE from *Nocardia asteroids* under optimized conditions (at 50 °C and pH 8) [236].

## 6. Role of Nanoparticles in Bioremediation

Despite its advantages, such as environmental friendliness and cost effectiveness, the traditional microbial-based bioremediation approach has some serious drawbacks that may reduce its efficacy. These include (1) less effectiveness in cleaning up extensive and highly polluted sites; (2) reduced efficacy in removing heavy metals, radioactive residues, and persistent organic pollutants; (3) excessive dependence on a number of environmental variables, including temperature, pH, and the availability of nutrients and oxygen; and (4) poor adaptation of pollutant-degrading micro-organisms to the pollutant compounds may result in a reduction in their degradation performance and bioavailability at contaminated sites [237]. In response to these constraints, a novel methodology known as “nano-bioremediation” has surfaced. This integrates biological processes with nanomaterials (non-biogenic or biogenic organic/inorganic nanoparticles) to achieve efficient, effective, and long-lasting remediation. They are manufactured solutions, utilizing several chemical processes (co-precipitation, co-reduction hydrothermal, and sol–gel) and biogenic methods, involving micro-organisms and plants [238]. Nanoparticles have garnered significant interest in diverse domains, such as bioremediation, owing to their distinct physical and chemical characteristics. Nevertheless, although they possess some benefits, there are certain obstacles linked to their utilization, such as evaluating their environmental fate and their possible effects on ecosystems and human health, as well as minimizing the expenses related to their manufacturing and functionalization.

Combining nanoparticles with bioremediation approaches has been shown to have synergistic benefits, with the combined strategy showing a more significant remediation effect than either method alone [239,240].

## 7. Combination of Nanoparticles and OP-Degrading Enzymes

Several innovative supports and techniques have recently emerged to augment traditional enzymatic immobilization with the aim of improving enzymatic loading, activity, and stability to minimize the cost of enzymatic biocatalysts in bioremediation processes. These methods encompass cross-linked enzymatic aggregations, microwave-assisted immobilization, and combining nanoparticles with microbial, insect, or plant enzymes (peroxidases and laccases) to enhance the effectiveness of bioremediation for various types of contaminants, including OPs (Table 2). Nanoparticles are gaining particular interest because of their distinctive physical and chemical characteristics, which include, among others, a large surface area-to-volume ratio, great mechanical strength [241], and great colloidal stability [242].

Wang et al. [243] studied the ability of a bacterial (*Pseudomonas aeruginosa*) recombinant organophosphorus hydrolase attached to mesoporous silica nanoparticles that are covered with a zwitterionic polymer to degrade methyl parathion. The enzyme was expressed in *E. coli* Rosetta (DE3) containing the plasmid pET-20b. It was reported that because of the zwitterionic polymer, which permitted methyl parathion enrichment onto the fabricated system, the immobilized enzyme exhibited a very low *K_m_* value (0.09 mM) in comparison to its free configuration (0.34 mM). The immobilized enzyme showed a catalytic efficiency (*k_cat_*/*K_m_*) of 17,367 s^−1^ mM^−1^, which was 2.4 times greater than that of the free enzyme (7221 s^−1^ mM^−1^). Moreover, the immobilized enzyme was able to maintain its stability for roughly 80% of its initial activity after 3 h at 40 °C and showed better pH tolerance and stability after multiple uses than the free enzyme.

In another study, an easy way to immobilize a recombinant organophosphorus hydrolase with His-tag obtained from *Brevundimonas diminuta* on organic-inorganic hybrid nanoparticles consisting of calcium phosphate nanocrystals and copper-modified bovine serum albumin was investigated. This facilitated the effective fabrication of a reusable, durable, and easily purifiable biocatalyst for breaking down methyl parathion. The immobilized enzyme showed improved stability in terms of pH and temperature compared to the enzyme in its free form. It displayed a higher residual activity value of about 60% at 60 °C compared to the free enzyme (only 20%) and showed a substantially superior tolerance to both alkaline and acidic conditions than the free enzyme. In addition, about 75% and 56% activities were shown by the immobilized enzyme after 5 and 10 times uses, respectively. Nevertheless, the immobilized biocatalyst displayed lower *k_cat_* (1767 min^−1^) and *k_cat_*/*K_m_* (5004 min^−1^ mmol^−1^ L) than the free enzyme (6362 min^−1^ and 11,822 min^−1^ mmol^−1^ L, respectively), probably due to adverse conformation modifications that may have affected the enzyme during the immobilization process [244].

Chen et al. [245] used the recombinant strain *E. coli* BL21 to produce PTE. The enzyme was mixed with CoCl_2_ and MnCl_2_ to form multi-metallic PTE hybrid nanoflowers, which were tested for their ability to degrade the pesticide methyl parathion and two chemical warfare agents (soman and nerve agent VX). More than 93% methyl parathion (190 µM) degradation was observed in a pump-flow reactor at a flow rate of 1 mL/min. Additionally, the *k_cat_*/*K_m_* value of the immobilized enzyme was 2.9 times higher than that of the free enzyme. The immobilized enzyme efficiently degraded 60 µM soman and 40 µM VX through hydroxyl nucleophilic attack within 60 min, releasing non-toxic products. On the other hand, it was observed that the produced nanoflowers showed better long storage and thermal stability and better pH, ionic concentration, inhibitors, and organic solvents tolerance when compared to the free enzyme, making them a good candidate for treating real OP-contaminated sites.

Das et al. [246] studied the degradation of chlorpyrifos by laccase from *Trametes versicolor* covalently immobilized onto iron oxide nanoparticles. The obtained laccase/magnetic iron nanoparticles were used in a 30-day laboratory-scale study to eliminate chlorpyrifos from soil that had been artificially contaminated. After the experiment, it was shown that the immobilized laccase was three times more efficient in removing the pesticide when compared with the control (without the enzyme). Also, it was suggested that the presence of copper in the fabricated biocatalyst could act by both adsorption and degradation during chlorpyrifos elimination in soil. This study demonstrated the potential of using laccase-immobilized iron nanoparticles for bioremediation of chlorpyrifos-contaminated soils.

**Table 2:** OP removal using enzyme immobilization on nanoparticles.

Enzyme/Source	OP	Carriers Used in the Immobilization Process	Efficiency	Ref
OP hydrolase	Methyl paraoxon	Poly-β-cyclodextrin microparticles	100%	[247]
PTE	Methyl parathion	Cu^2+^-based enzyme hybrid nanoflowers	62.5%	[248]
OP hydrolase from *Flavobacterium* sp. ATCC 27551	Ethyl parathion	Two types of cellulose microfibers produced by using chemical coupling agents (1,4-butanediol diglycidyl ether and 1,1′-Carbonyldiimidazole)	68% and 73%	[249]
OP hydrolase	Methyl parathion	Yolk-shell structured Co/C@SiO_2_@Ni/C nano-composites based MOFs ZIF-67 coated with PDA-Ni^2+^ shell	100%	[250]
PTE	Paraoxon	DNA cage-semiconductor quantum dot nanocomposites	~100%	[251]
Laccase from *Bacillus* sp.	Chlorpyrifos	Iron magnetic nanoparticles (Fe_3_O_4_)		[252]
OP hydrolase from *Flavobacterium* sp. ATCC 27551	Ethyl paraoxon	Magnetosomes of magnetite (Fe_3_O_4_)	100%	[253]
OP hydrolase	Paraoxon	Mesoporous silica nanoparticles	100%	[254]
Carboxylesterase from *Spodoptera litura*	Malathion	Mesoporous silica nanoparticles (SBA-15)		[255]

## 8. Conclusions

Enzymes from micro-organisms that stoichiometrically neutralize or degrade OPs with a turnover can be isolated and purified from natural sources. Constant efforts have been made in these directions for more than 30 years. Then, recombinant enzymes can be easily produced, using simple prokaryotic expression systems (e.g., *E. coli*). Engineering improvement of catalytic activity toward the large spectrum of OPs is the main task for both medical and environmental uses. The spectrum of OPs can also be expanded by combining several engineered enzymes in bioscavenger “cocktails” [256]. Several additional issues are mandatory for efficiency, safety, and economic reasons. Increase in enzyme conformational stability for long-term storage in solution or in dry forms and improvement of in vivo operational stability and immunotolerance are important goals for the medical uses of these enzymes. All these tasks imply genetic, chemical, and physical engineering of enzymes. The different strategies can be implemented. The research of new natural enzymes in collections of bacterial strains [257], in extreme environments [258] and identification (mining) of enzymes from genomic sequence databases is the first task. In the past ten years, this approach has been extremely fruitful. In particular, about mining, new enzyme DNA sequences from extremophilic PLL were identified, genes were synthesized and enzymes expressed in a mesophilic bacterial host, and then catalytic properties and X ray structure were determined [120,132]. Thus, research of enzymes of interest by computational structure mining in PDB database is extremely promising [259]. Also, because extremophile micro-organisms have a great potential, exploration of extreme biotopes is of the utmost importance. For example, novel extremozymes, PLL and PROL, have been discovered by screening halophilic, hyperthermophilic, piezophilic, radioresistant bacteria and archaeas in such extreme environments. Engineering of novel enzymes is the next task. Site-directed mutagenesis and directed evolution approaches in combination with chemical modifications and medium manipulations have been successfully used to improve the desired properties, in particular stereo-selectivity, high *k_cat_*/*K_m_*, and broad spectrum of activity of PTEs and stability [208,260,261]. Humanization of microbial enzymes is another possible engineering strategy. In particular, it should be noted that a human PROL, showing sequence homologies with *Alteromonas haloplanktis* PROL, displays a catalytic activity against sarin and soman [262]. Thus, humanization of this bacterial PROL by genetic engineering could be a way to produce safe and effective recombinant PROLs to be used as catalytic bioscavengers.

Alternatively, computational re-design (molecular modeling, transition state simulations, and QM/MM approaches) of known enzymes is another emerging fruitful strategy. The development of artificial intelligence following the progress of in silico approaches is expected to lead to new mutated enzymes with higher activity against wider ranges of OPs.

Thus, all implemented and integrated strategies are progressively leading to more effective enzymes with improved physical and pharmaceutical properties and allowing production at a lower cost. The cost of enzyme production is certainly the main limiting factor, and considerable efforts have to be made to reduce it. For medical applications, various formulations of catalytic bioscavengers have already been used for skin protection, decontamination, and safe prophylaxis and post-exposure treatments of OP poisoning. Multiple enzyme formulations will extend the activity spectrum of free or encapsulated enzymes in nanoparticles or in nanoreactors. Moreover, new gene therapy approaches may offer the possibility of the transitory production of humanized bacterial OP-degrading enzymes in the body. However, besides ethical issues, more research works are still needed to engineer safe gene therapy vectors that do not produce toxic viral proteins and/or induce adverse immune responses.

As for applications to environmental decontamination and remediation, the sustainable and environmentally friendly approach using enzymes for OPs degradation holds immense potential for remediation efforts and reducing the environmental impact of these toxic substances. In addition, OP-degrading enzymes can be immobilized on various supports and matrices, allowing for their reuse multiple times. This decreases costs and increases the overall performance of the biocatalysts. Although OP-degrading enzymes have shown significant success in in vitro studies, their use in real bioremediation conditions needs to overcome some drawbacks, such as their unstable efficiency, their vulnerability to organic and inorganic inhibitors, and their ineffectiveness against some OPs, such as V-type nerve agents, rendering more research necessary to resolve these issues. Future research efforts will probably focus further on using engineered OP-degrading enzymes alone or encapsulated in nanoparticles to attain better degradation and stability properties. For example, significant thermostability improvement of OP-degrading enzymes could be achieved with engineering techniques such as flexible loop truncation, proline substitutions, ionic pair networks creation [263], and self-assembling amphipathic peptides fusion [264].

## Figures and Tables

**Table 1:** The most investigated microbial OP-degrading enzymes.

	Type of Organism	Enzyme	Ref.
Bacteria	*Agrobacterium radiobacter*	OpdA (organophosphate hydrolase)	[50]
	*Bacillus thuringiensis* MB497	OPP (organophosphorus phosphatase)	[51]
	*Alteromonas* sp., *Alteromonas haloplanktis*, *Alteromonas undin*	OPAA (organophosphorus acid anhydrolase)	[19]
	*Flavobacterium* sp.	OPD (organophosphate hydrolase)	[52]
	*Achromobacter xylosoxidans* GH9OP, *Arthrobacter* sp. HM01, *Brevundimonas diminuta*.	OPH/OpdH (organophosphosphate hydrolase)/PTE (phospho-triesterase)/aryl-dialkyl-phosphatase	[49,52,53]
	*Cronobacter muytjensii* GH10, *Pseudaminobacter* sp. mp-1, *Pseudomonas aeruginosa* GH2NO8, *Brevundimonas diminuta* MG (formely *Pseudomonas diminuta* MG), *Pseudomonas monteilii* C11		
	*Pseudomonas* sp. WBC-3, *Plesiomonas* sp. M6	MPH (methyl parathion hydrolase)	[50,52]
Engineered	Engineered *Escherichia coli*	OPH-E (organophosphosphate hydrolase)/parathion hydrolase	[54]
Archaebacteria	*Sulfolobus solfataricus* *Vulcanisaeta moutnovskia*	Ssopox Phosphotriesterase-like lactonase	[55] [56]
Fungi	*Caldariomyces fumago*	Chloroperoxidase	[57]

## Abbreviations

AChE: acetylcholinesterase; BChE, butyrylcholinesterase; CaE, carboxylesterase; ChE, cholinesterase; CWA, chemical warfare agent; DFP, diisopropylfluorophosphate; OP, organophosphates, organophosphorous; OPAA, organophosphoric acid anhydrolase; OPAH, organophosphorus acid anhydride hydrolase; OPH, OPase organophosphate hydrolase; PLL, phosphotriesterase-like lactonase; PON-1, paraoxonase; PTE, phosphotriesterase.
